# Supplementary material for: AAV11 enables efficient retrograde targeting of projection neurons and enhances astrocyte-directed transduction
Source: Nat Commun. 2023 Jun 26;14:3792. doi: 10.1038/s41467-023-39554-7 (PMC10293207; doi:10.1038/s41467-023-39554-7)
Supplement: Supplementary file 1 — Supplementary Information [file 41467_2023_39554_MOESM1_ESM.pdf]

AAV11 enables efficient retrograde targeting of projection neurons and enhances astrocyte-directed transduction

Zengpeng Han[1,2,3,4,#], Nengsong Luo[5,#], Wenyu Ma[2,4], Xiaodong Liu[6], Yuxiang Cai[5], Jiaxin Kou[7], Jie Wang[2,4], Lei Li[2], Siqi Peng[8], Zihong Xu[8], Wen Zhang[8], Yuxiang Qiu[1,3,4], Yang Wu[2,4], Chaohui Ye[2,4], Kunzhang Lin[1,3] * & Fuqiang Xu[1,2,3,4,5,9] *

Corresponding author e-mails: Kunzhang Lin (kz.lin@siat.ac.cn.)

Fuqiang Xu (fq.xu@siat.ac.cn)

Supplementary Information

(Contains Supplementary Figures 1-14 with Legends)

Supplementary Information

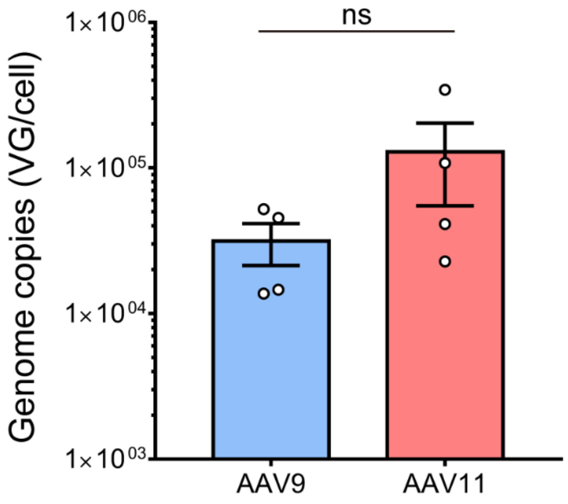

Supplementary Fig. 1 AAV11 produces high yields equivalent to AAV9. The recombinant AAV11 or AAV9 were obtained by co-transfection of three plasmids into HEK-293T cells. Both cell lysate and media were pooled for titer determination and calculation of average yield per cell. Statistical values are indicated as mean ± SEM (n = 4/group); Statistical analyses were performed using an unpaired two-tailed Student's t test, with significant differences being expressed by the p value, ns, no significant difference was observed. $p > 0.05$. Source data are provided as a Source Data file.

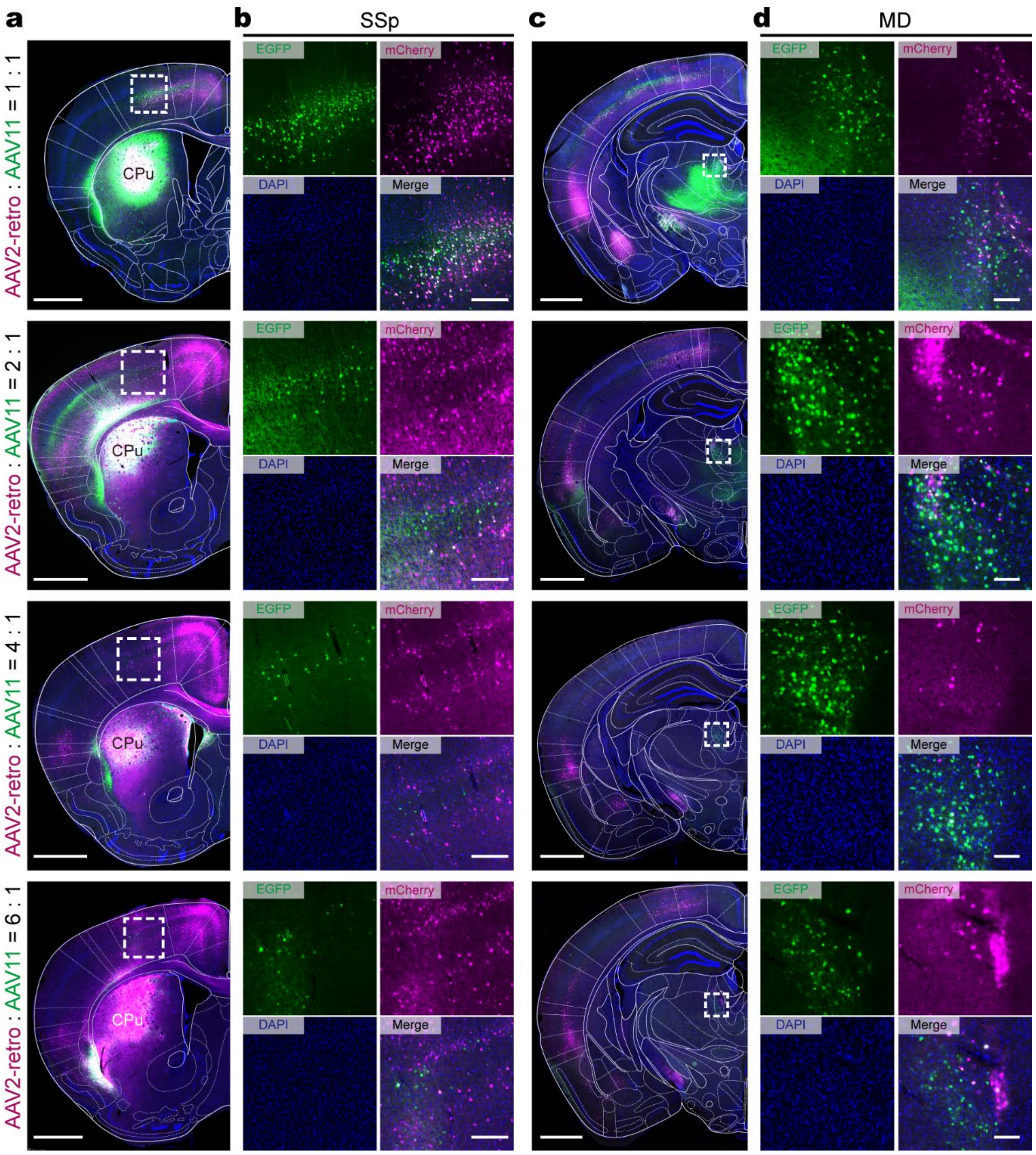

Supplementary Fig. 2 Retrograde transduction of AAV11 and AAV2-retro through mixed injection with different dose gradients into the CPu. a The fluorescence distribution of EGFP at the injection site of caudate-putamen (CPu) under injection with different dose gradients of viruses. AAV2-retro-EF1α-mCherry and AAV11-EF1α-EGFP were mixed at different particle ratios of 1:1, 2:1, 4:1 and 6:1 (3×10^9 VG in total, 300 nL) and injected respectively into CPu of C57BL/6 mice, *n* = 3/group. Scale bar = 1 mm. **b** Magnified partial view of SSp region in figure a. Scale bar = 200 μm. **c** Fluorescence distribution of EGFP (AAV11) and mCherry (AAV2-retro) in upstream regions of CPu. Scale bar = 1 mm. **d** Magnified partial view of MD region in figure c. Scale bar = 100 μm.

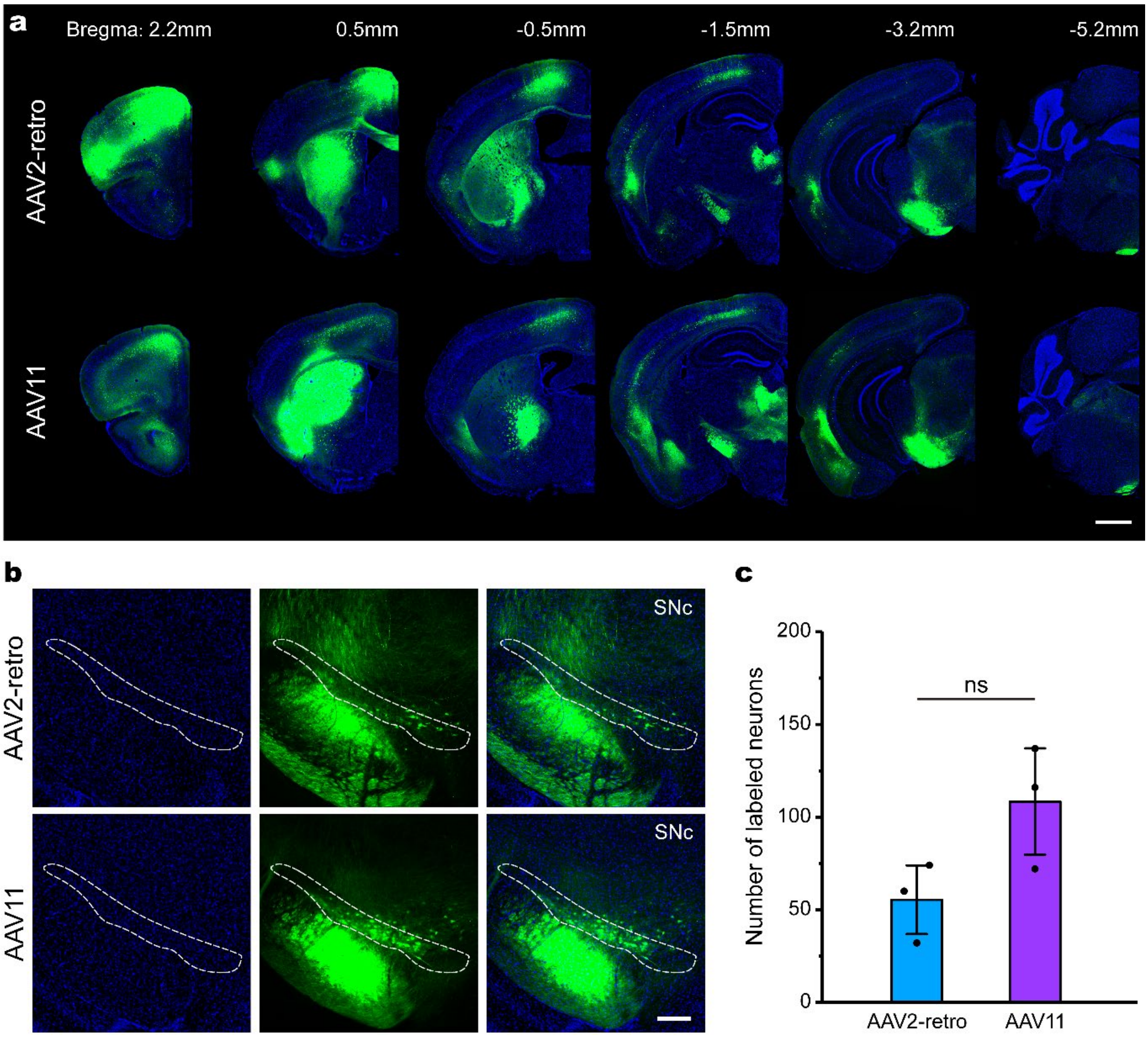

Supplementary Fig. 3 Retrograde targeting with AAV11 and AAV2-retro driven by a strong promoter. a 200 nL each of AAV11 and AAV2-retro (4 × 10^9 VG each virus) driven by the CAG promoter were injected into CPu area. The EGFP signals in anteroposterior slices of CPu area were imaged at 21 days post-injection. Scale bar = 1 mm. **b** AAV11- and AAV2-retro-infected projection neurons in compact part of substantia nigra (SNc). **c** Quantification of AAV11- and AAV2-retro-infected projection neurons. Statistical values are presented as mean ± SEM (n = 3/group). Statistical analyses were performed using an unpaired two-tailed Student's t test, with significant differences being expressed by the p value. No significant difference was observed, $p > 0.05$. Source data are provided as a Source Data file.

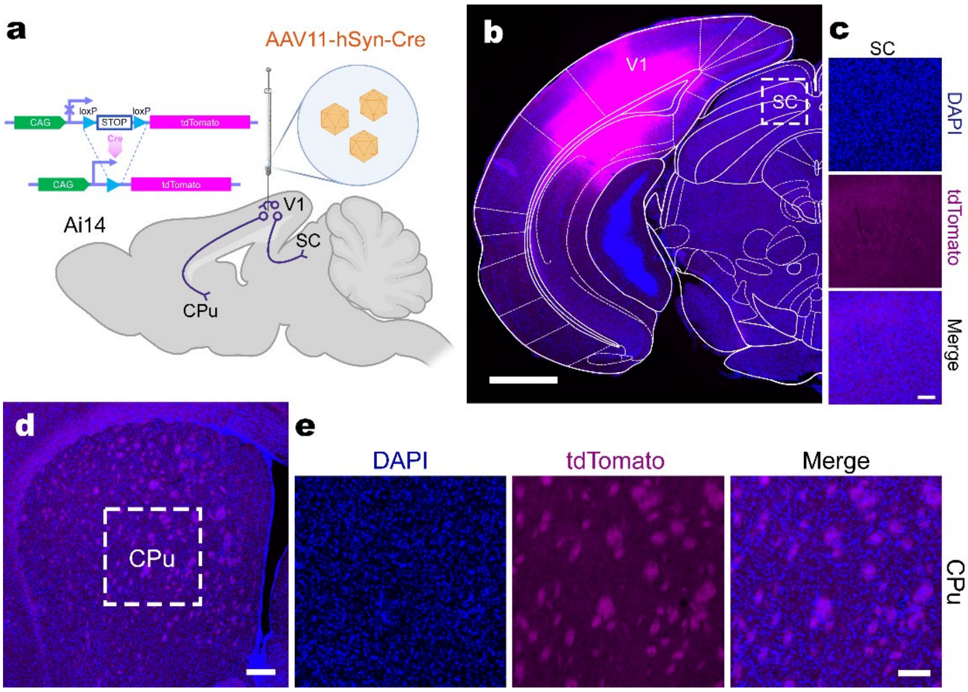

Supplementary Fig. 4 AAV11 does not exhibit anterograde transsynaptic propagation properties in circuits of V1 to SC and V1 to CPu. **a** Schematics of the injection site (primary visual cortex, V1) and the connected downstream areas such as superior colliculus (SC) and caudate-putamen (CPu). AAV11-hSyn-Cre (2 × 10^9 VG per mouse, 200 nL, *n* = 3) was injected into Ai14 transgenic mice, in which the expression of tdTomato fluorescent reporter is Cre-dependent. Diagram was created with BioRender.com. **b** Expression of tdTomato after injection of AAV11-hSyn-Cre into the V1 of Ai14 transgenic mice. No tdTomato-positive neurons were detected in the SC. Scale bar = 1 mm. **c** Magnified partial view of SC region in figure b. Scale bar = 1 mm. **d** No tdTomato-positive neurons were detected in the CPu. Scale bar = 200 μm. **e** Magnified partial view of CPu region in figure d. Scale bar = 100 μm.

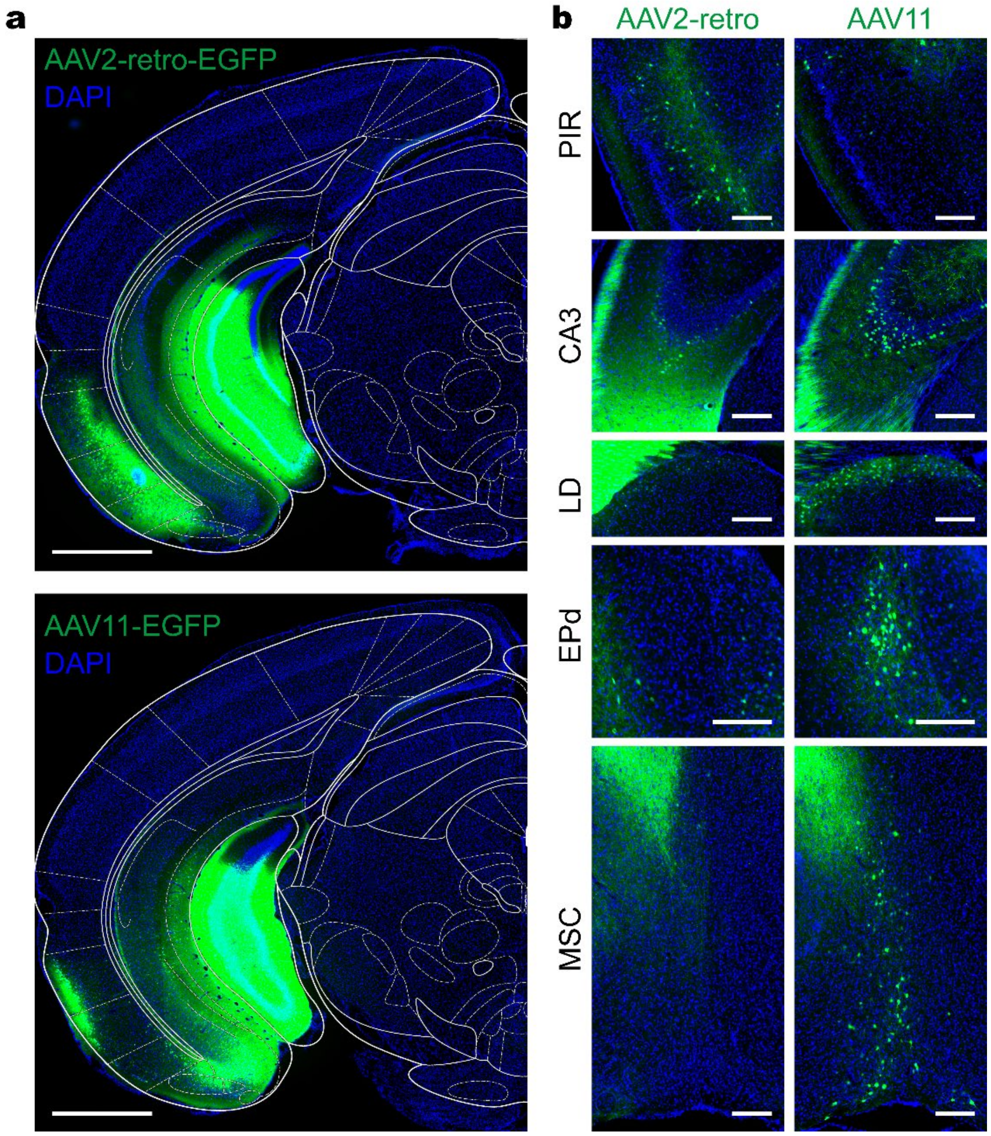

Supplementary Fig. 5 Comparison of the retrograde transduction tropism of AAV11 and AAV2-retro following independent injections into vHPC. a Fluorescence distribution of EGFP at the injection site, AAV2-retro-EF1α- EGFP and AAV11-EF1α-EGFP viruses were injected into vHPC of C57BL/6 mice (3×10^9 VG per mouse, 300 nL, n = 3). Scale bar = 1 mm. **b** Representative images reveal that the retrograde labeling efficiencies of AAV11 and AAV2-retro are quite different in many regions, such as the piriform area (PIR), hippocampal field CA3 (CA3), lateral dorsal nucleus of thalamus (LD), endopiriform nucleus dorsal part (EPd), and medial septal complex (MSC). Scale bar = 200 μm.

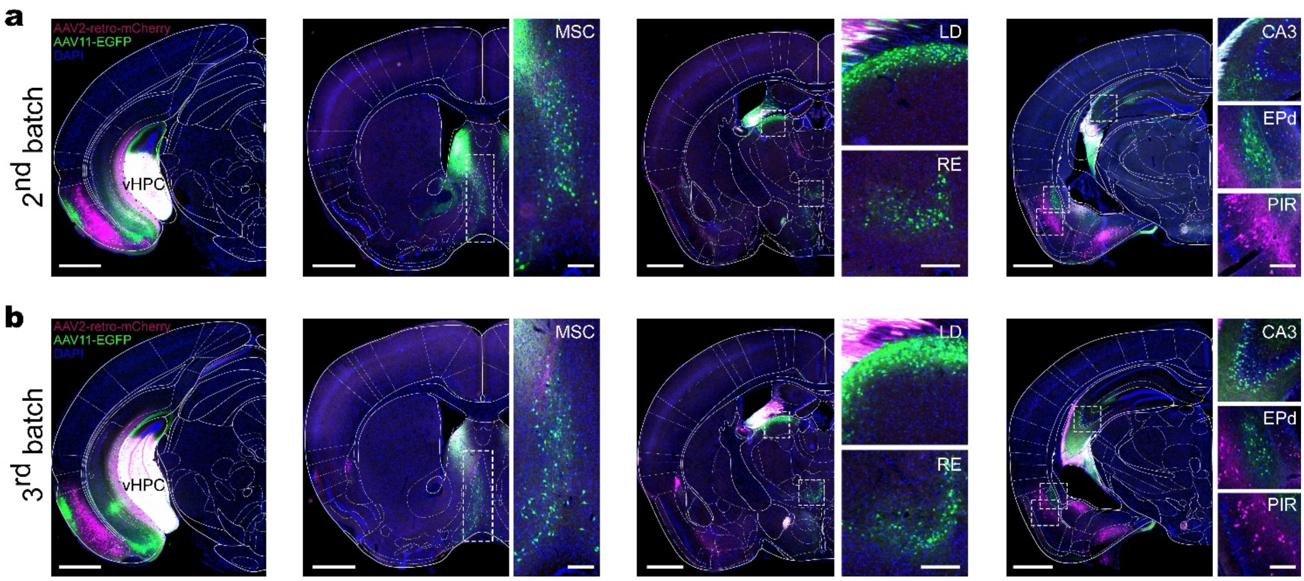

Supplementary Fig. 6 Comparison of different batches of both AAV11 and AAV2-retro in retrograde transduction tropism after vHPC infusion. a After the second batch of virus preparation, the AAV11-EF1α-EGFP (green) and AAV2-retro-EF1α-mCherry (magenta) viruses were mixed at a 1:1 particle ratio and injected into the vHPC brain region of C57BL/6 mice (3 × 10^9 VG in total, 300 nL per mouse, *n* = 3/group), and many upstream brain regions were labeled, such as medial septal complex (MSC), lateral dorsal nucleus of thalamus (LD), nucleus of reuniens (RE), hippocampal field CA3 (CA3), endopiriform nucleus dorsal part (EPd), and piriform area (PIR). Scale bar = 1 mm for the large pictures on the left and 200 μm for magnified partial view. **b** The prepared third batch of viruses have similar labeling effect to the second batch of viruses. Scale bar = 1 mm for the large pictures on the left and 200 μm for magnified partial view.

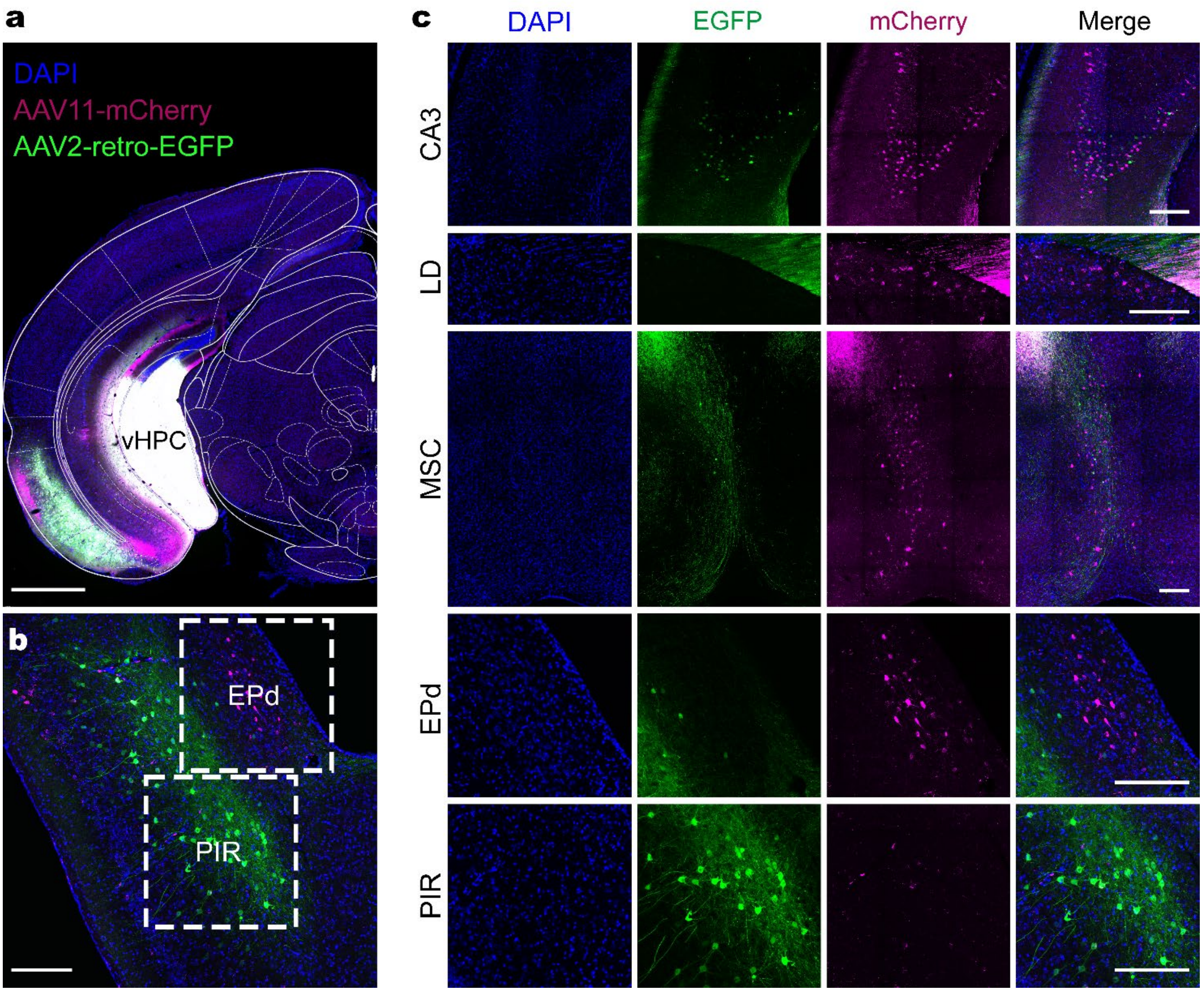

Supplementary Fig. 7 Comparison of AAV11 and AAV2-retro in retrograde transduction tropism after vHPC infusion with exchange of the carried fluorescent gene. a Fluorescence distribution of EGFP (AAV2-retro, green) and mCherry (AAV11, magenta) at the vHPC injection site. AAV2-retro-EF1α-EGFP-WPRE-pA and AAV11-EF1α-mCherry-WPRE-pA were mixed at a particle ratio of 1:1 (3×10^9 VG in total, 300 nL per mouse, n = 3) and injected into vHPC of C57BL/6 mice. Scale bar = 1 mm. **b, c** Representative images reveal that the retrograde labeling efficiencies of AAV11-EF1α-mCherry and AAV2-retro-EF1α-EGFP are quite different in many regions, such as hippocampal field CA3 (CA3), lateral dorsal nucleus of thalamus (LD), medial septal complex (MSC), endopiriform nucleus dorsal part (EPd) and piriform area (PIR). Scale bar = 200 μm.

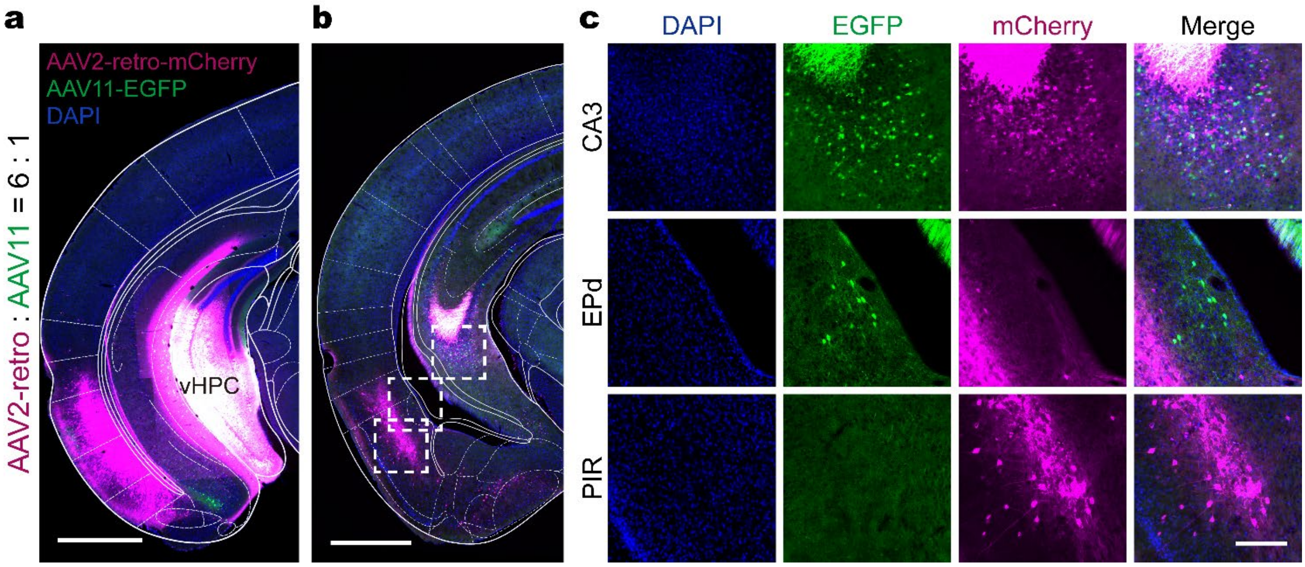

Supplementary Fig. 8 Comparison of AAV11 and AAV2-retro in retrograde transduction tropism after vHPC infusion with AAV11 administered in low doses. a Fluorescence distribution of EGFP (AAV11, green) and mCherry (AAV2-retro, magenta) at the vHPC injection site. AAV2-retro-EF1α-mCherry-WPRE-pA and AAV11-EF1α-EGFP-WPRE-pA were mixed at a particle ratio of 6:1 (1×10^9 VG in total, 300 nL per mouse, n = 3) and injected into vHPC of C57BL/6 mice. Scale bar = 1 mm. **b** Fluorescence distribution of EGFP (AAV11) and mCherry (AAV2-retro) in upstream regions of vHPC, such as hippocampal field CA3 (CA3), endopiriform nucleus dorsal part (EPd) and piriform area (PIR). Scale bar = 1 mm. **c** Magnified partial view of CA3, EPd and PIR region in figure b. Scale bar = 200 μm.

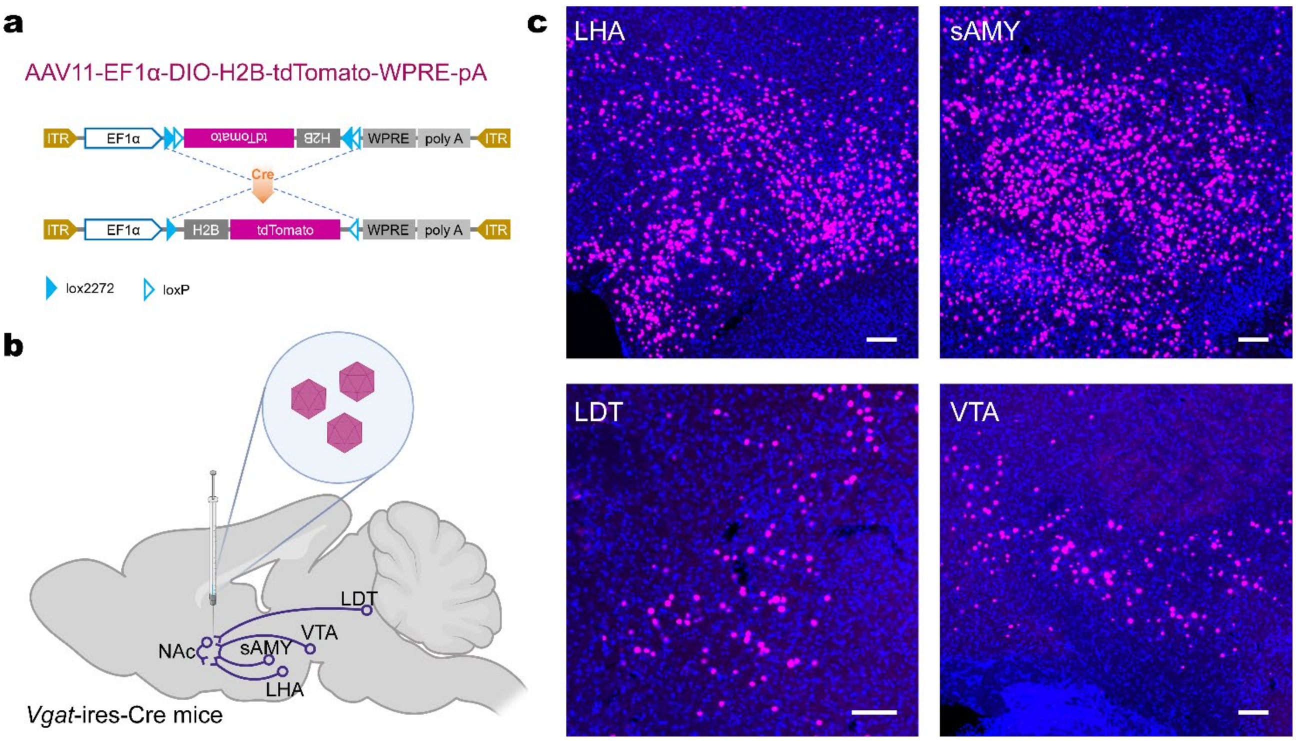

Supplementary Fig. 9 Combination of AAV11 with Cre transgenic lines for retrograde targeting of genetically defined neuronal populations. a Schematic digram of viral vector carrying a Cre-dependent nuclear-localized tdTomato. DIO, double-floxed inverted orientation. **b** Schematic diagram of virus injection. AAV11 (2×10^9 VG per mouse, n = 3) was injected into NAc of *Vgat*-ires-Cre mice. Diagram was created with BioRender.com. **c** Fluorescence was restricted to GABAergic neurons-specific projection neurons in lateral hypothalamic area (LHA), striatum-like amygdalar nuclei (sAMY), laterodorsal tegmental nucleus (LDT), and ventral tegmental area (VTA). Scale bar = 100 μm.

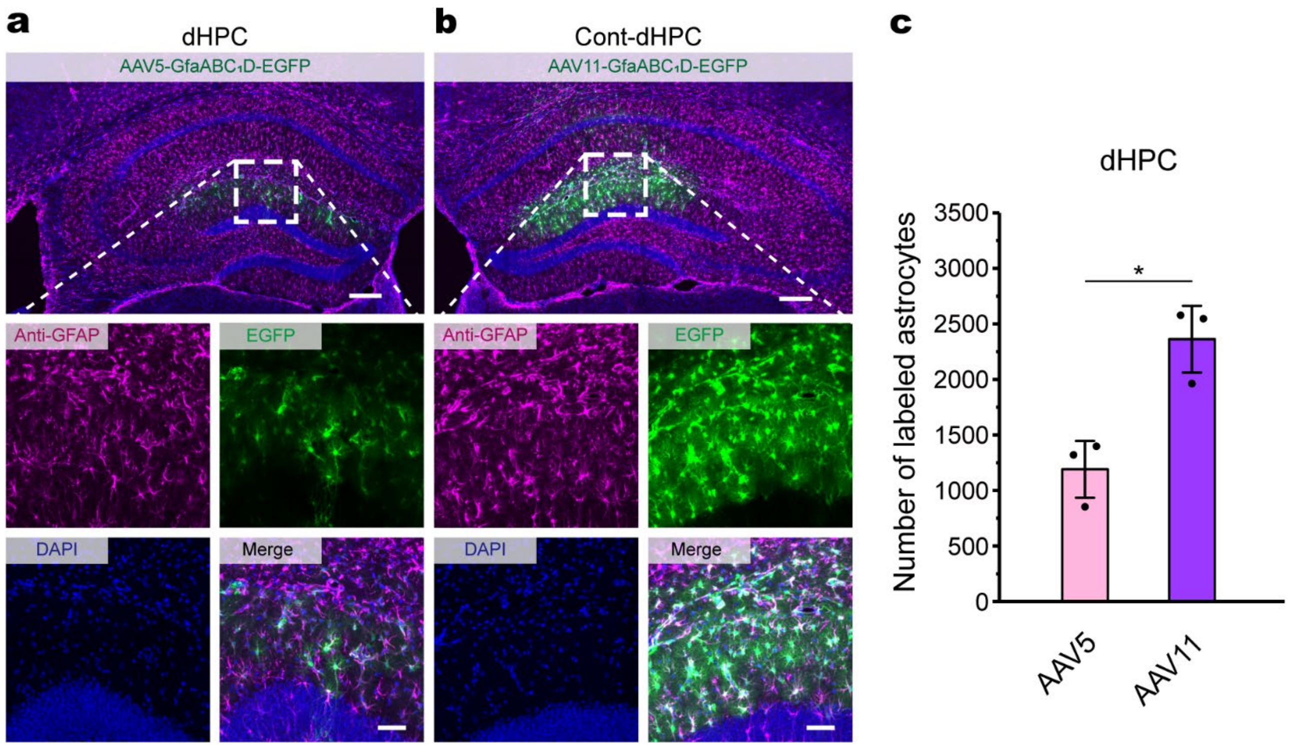

Supplementary Fig. 10. Comparison of AAV11 and AAV5 on astrocyte-specific transduction in dHPC. a, b Representative images of dorsal hippocampus (dHPC) astrocytes infected by AAV5 (a, left) and AAV11 (b, right). 100 nL each of AAV11-GfaABC$_1$D-EGFP and AAV5-GfaABC$_1$D-EGFP (5×10^8 VG each virus, n = 3/group) were injected into the right and left sides of the dHPC, respectively. The sections stained with antibody against GFAP (magenta) show the distribution of astrocytes. EGFP signals (green) colocalizing with GFAP staining (magenta) indicate the transduced astrocytes (appeared in white). Cont-dHPC: contralateral dHPC. Scale bar = 200 μm (top 2 panels), 50 μm (bottom 8 panels). **c** Quantification of astrocyte-specific transduction in the indicated regions. Statistical values are presented as mean ± SEM (n = 3/group). Statistical analyses were performed using an unpaired two-tailed Student's t test, with significant differences being expressed by the p value. *$p < 0.05$. Source data are provided as a Source Data file.

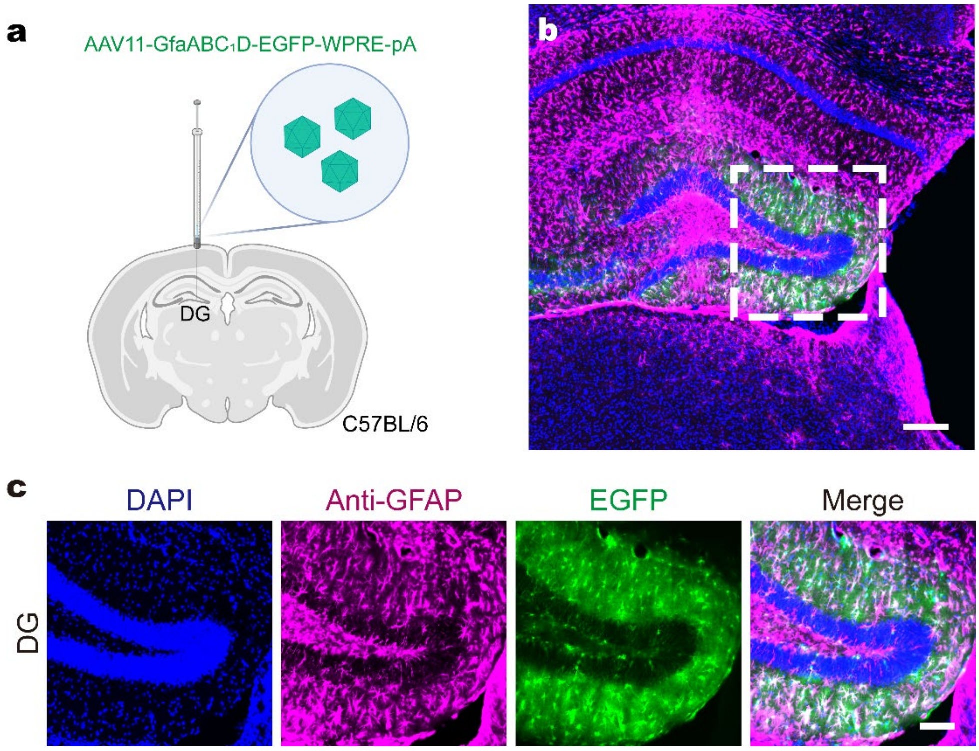

Supplementary Fig. 11 AAV11 enables astrocytes transduction in hippocampal dentate gyrus. a Schematic diagram of virus injection, AAV11-GfaABC$_1$D-EGFP (2 × 10^9 VG per mouse) was injected into dentate gyrus (DG) area of C57BL/6 mice, n = 3. Diagram was created with BioRender.com. **b** EGFP expression at the injection site of DG. GFAP immunoreactivity indicates astrocytes. Scale bar = 200 μm. **c** Magnified partial view of DG region in figure b. Scale bar = 100 μm.

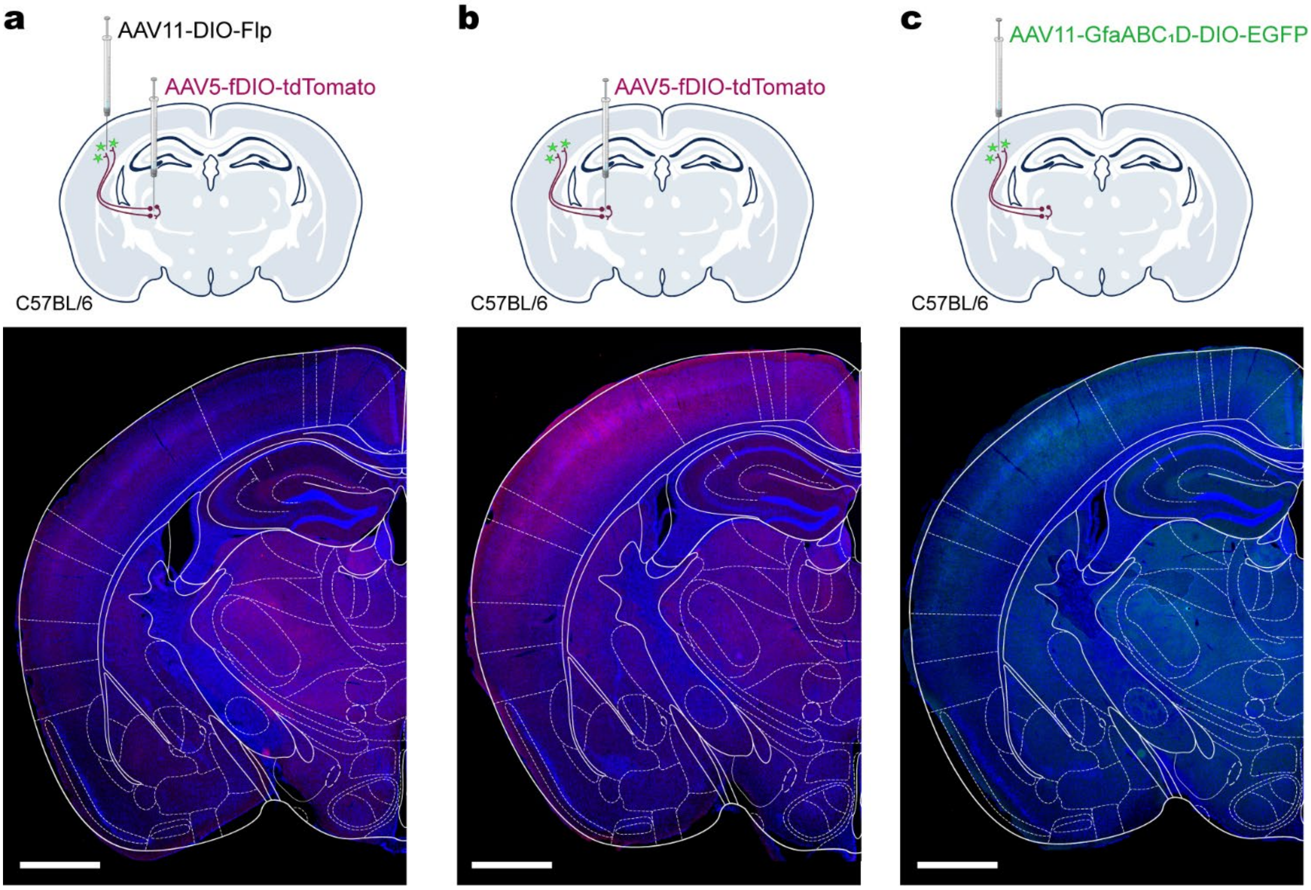

Supplementary Fig. 12 Leaking test of recombinase-dependent vectors. a AAV5-EF1α-fDIO-tdTomato was injected into the VPM (1.5×10^9 VG in total, 150 nL, *n* = 3), and AAV11-EF1α-DIO-Flp were injected into BX (1.5×10^9 VG in total, 150 nL, *n* = 3). No red fluorescence signals were observed at the injection site. **b** AAV5-EF1α-fDIO-tdTomato was injected into the VPM (1.5×10^9 VG in total, 150 nL, *n* = 3). No red fluorescence signals were observed at the injection site. **c** AAV11-GfaABC$_1$D-DIO-EGFP was injected into BX (1.5×10^9 VG in total, 150 nL, *n* = 3). No green fluorescence signals were observed at the injection site. Schematic diagrams were created with BioRender.com.

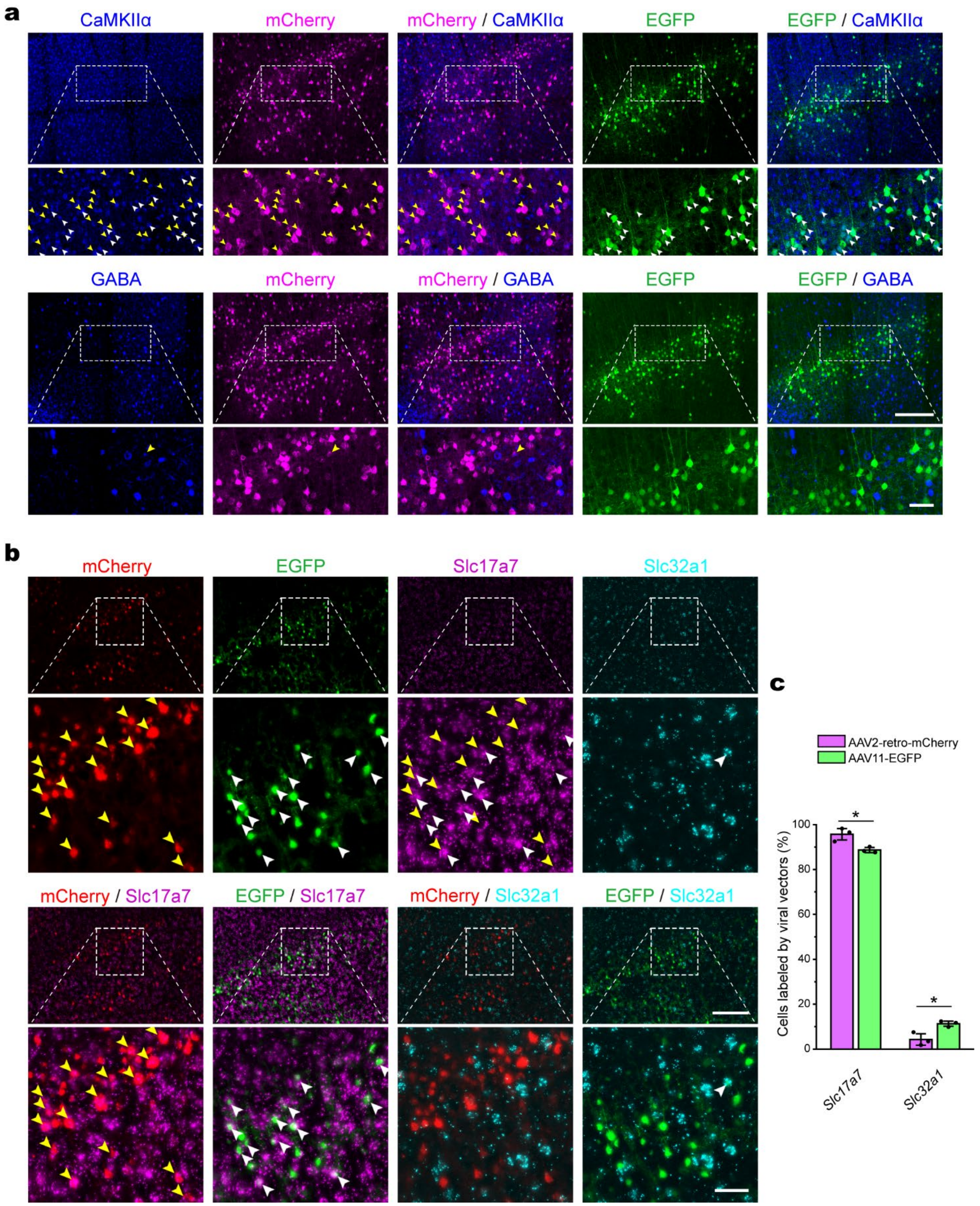

Supplementary Fig. 13 Identification of cell types labeled by viral vectors in the SSp brain region using immunofluorescence or *in situ* hybridization. a Fluorescence distribution by immunofluorescence in SSp brain region. AAV2-retro-EF1α-mCherry

(magenta) and AAV11-EF1α-EGFP (green) were mixed and injected respectively into CPu of C57BL/6 mice (3×10^9 VG in total, 300 nL, *n* = 3/group). The blue fluorescence signals indicate CaMKIIα protein (an excitatory neuron marker) in the top panel, and GABA protein (an inhibitory neuron marker) in the bottom panel. Yellow (co-labeled with mCherry) or white (co-labeled with EGFP) arrows indicate the location of cells with co-labeled signals. Scale bar = 200 μm for the large pictures and 50 μm for magnified partial views. **b** Fluorescence distribution by *in situ* hybridization in SSp brain region. AAV2-retro-EF1α-mCherry (red) and AAV11-EF1α-EGFP (green) were mixed injected respectively into CPu of C57BL/6 mice (3×10^9 VG in total, 300 nL, *n* = 3/group). The magenta fluorescence signals indicate *Slc17a7* (an excitatory neuron marker) and the cyan fluorescence signals indicate *Slc32a1* (an inhibitory neuron marker). Yellow (co-labeled with mCherry) or white (co-labeled with EGFP) arrows indicate the location of cells with co-labeled signals. Scale bar = 200 μm for the large pictures and 50 μm for magnified partial views. **c** Analysis of the colocalization of viral vector's reporter signals and targeting marker of *in situ* hybridization. Statistical values are presented as mean ± SEM (*n* = 3/group). Statistical analyses were performed through unpaired two-tailed Student's *t* tests, with significant differences being expressed by the *p* value. *$p < 0.05$. Source data are provided as a Source Data file.

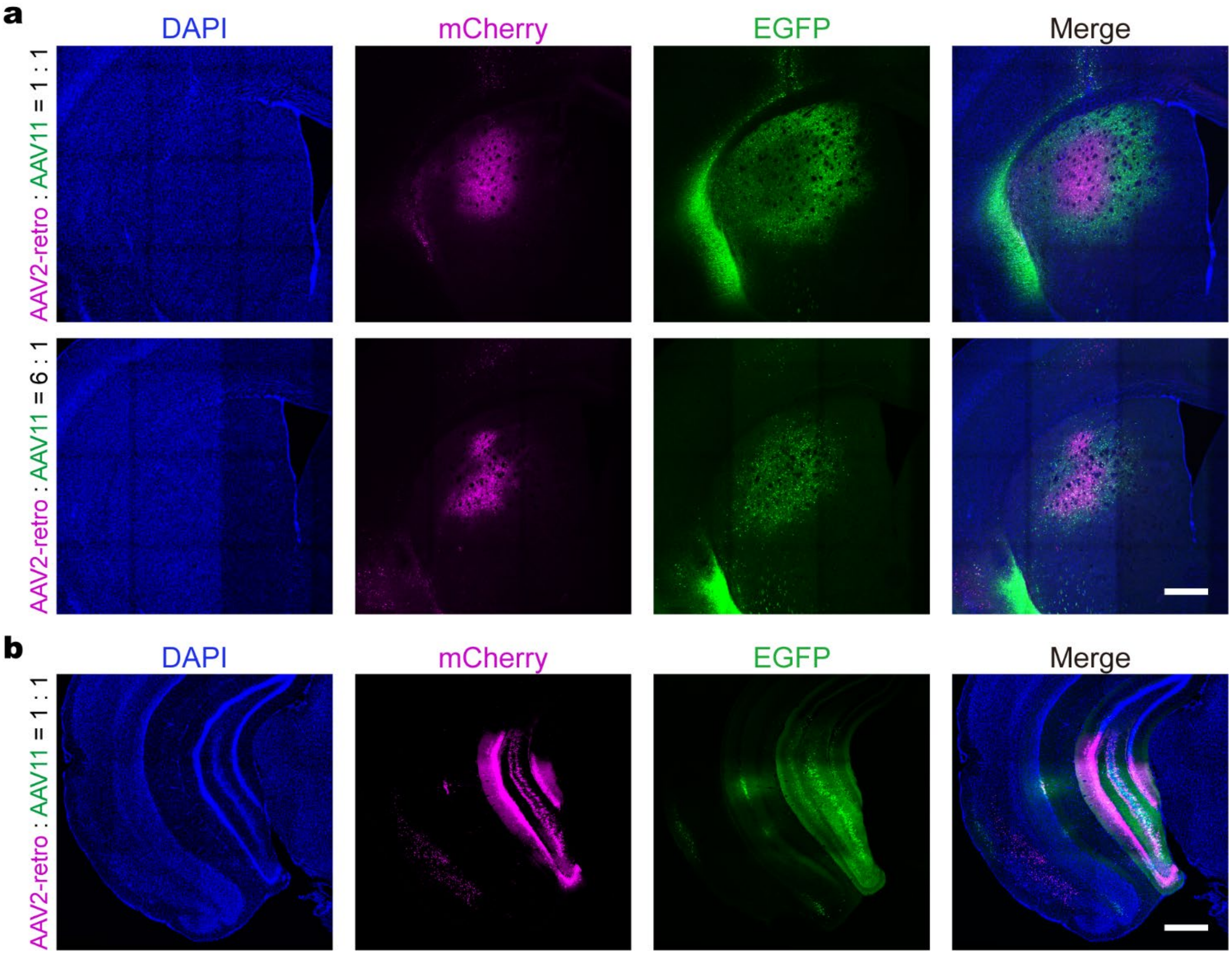

Supplementary Fig. 14 The *in situ* transduction of viral vectors. a Fluorescence distribution of mCherry (AAV2-retro, magenta) and EGFP (AAV11, green) at the CPu injection site. *n* = 3/group. Scale bar = 1 mm. **b** Fluorescence distribution of mCherry (AAV2-retro, magenta) and EGFP (AAV11, green) at the vHPC injection site. *n* = 3/group. Scale bar = 1 mm.
