# Supplementary material for: AAV11 enables efficient retrograde targeting of projection neurons and enhances astrocyte-directed transduction
Source: Nat Commun. 2023 Jun 26;14:3792. doi: 10.1038/s41467-023-39554-7 (PMC10293207; doi:10.1038/s41467-023-39554-7)
Supplement: Supplementary file 3 — Reporting Summary [file 41467_2023_39554_MOESM3_ESM.pdf]

nature portfolio

Corresponding author(s): Kunzhang Lin and Fuqiang Xu

Last updated by author(s): Jun 8, 2023

Reporting Summary

Nature Portfolio wishes to improve the reproducibility of the work that we publish. This form provides structure for consistency and transparency in reporting. For further information on Nature Portfolio policies, see our Editorial Policies and the Editorial Policy Checklist.

Statistics

For all statistical analyses, confirm that the following items are present in the figure legend, table legend, main text, or Methods section.

n/a	Confirmed	
☐	☒	The exact sample size (n) for each experimental group/condition, given as a discrete number and unit of measurement
☐	☒	A statement on whether measurements were taken from distinct samples or whether the same sample was measured repeatedly
☐	☒	The statistical test(s) used AND whether they are one- or two-sided<br>*Only common tests should be described solely by name; describe more complex techniques in the Methods section.*
☒	☐	A description of all covariates tested
☒	☐	A description of any assumptions or corrections, such as tests of normality and adjustment for multiple comparisons
☐	☒	A full description of the statistical parameters including central tendency (e.g. means) or other basic estimates (e.g. regression coefficient) AND variation (e.g. standard deviation) or associated estimates of uncertainty (e.g. confidence intervals)
☐	☒	For null hypothesis testing, the test statistic (e.g. F, t, r) with confidence intervals, effect sizes, degrees of freedom and P value noted<br>*Give P values as exact values whenever suitable.*
☒	☐	For Bayesian analysis, information on the choice of priors and Markov chain Monte Carlo settings
☒	☐	For hierarchical and complex designs, identification of the appropriate level for tests and full reporting of outcomes
☒	☐	Estimates of effect sizes (e.g. Cohen's d, Pearson's r), indicating how they were calculated

Our web collection on statistics for biologists contains articles on many of the points above.

Software and code

Policy information about availability of computer code

Data collection	Leica TCS SP8 confocal microscope (Leica, TCS SP8, Leica, Wetzlar, Germany) or the Olympus VS120 Slide Scanner microscope (Olympus, Tokyo, Japan) for imaging, fiber photometry system (ThinkerTech, Nanjing, Jiangsu, China) for calcium transients recording.
Data analysis	GraphPad Prism 7.00, Origin 7.0, MATLAB R2018b, Microsoft Excel and ImageJ 1.8.0.

For manuscripts utilizing custom algorithms or software that are central to the research but not yet described in published literature, software must be made available to editors and reviewers. We strongly encourage code deposition in a community repository (e.g. GitHub). See the Nature Portfolio guidelines for submitting code & software for further information.

Data

Policy information about availability of data

All manuscripts must include a data availability statement. This statement should provide the following information, where applicable:

- Accession codes, unique identifiers, or web links for publicly available datasets
- A description of any restrictions on data availability
- For clinical datasets or third party data, please ensure that the statement adheres to our policy

The authors declare that all supporting data for this study are included within the manuscript and its Supplementary Information files or can be obtained from the authors upon reasonable request. The nucleic acid sequence for AAV11 is accessible through the NCBI GenBank (accession number: AY631966.1). The relevant source data, including those for Figs. 2-7 and Supplementary Figs. 1, 3, 10, 13, are consolidated in the Source Data file accompanying this paper.

Human research participants

Policy information about studies involving human research participants and Sex and Gender in Research.

Reporting on sex and gender	N/A
Population characteristics	N/A
Recruitment	N/A
Ethics oversight	N/A

Note that full information on the approval of the study protocol must also be provided in the manuscript.

Field-specific reporting

Please select the one below that is the best fit for your research. If you are not sure, read the appropriate sections before making your selection.

☒ Life sciences ☐ Behavioural & social sciences ☐ Ecological, evolutionary & environmental sciences

For a reference copy of the document with all sections, see nature.com/documents/nr-reporting-summary-flat.pdf

Life sciences study design

All studies must disclose on these points even when the disclosure is negative.

Sample size	The sample size was determined according to previous studies (PMID: 27720486, PMID: 31427575, PMID: 32606306). For experiments in mice, all the biological experiments were performed in three replicated or more.
Data exclusions	No data was excluded.
Replication	Each experimental test in the manuscript was independently repeated at least three times, with all repetitions yielding consistent results.
Randomization	For all non-animal studies, sample allocation was randomized. For in vivo studies, randomization was based on initial animal weight to equalize starting weights across groups.
Blinding	All experimental procedures and quantification of results, including cultures, injections, imaging, and statistical analysis were done by three independent researchers.

Reporting for specific materials, systems and methods

We require information from authors about some types of materials, experimental systems and methods used in many studies. Here, indicate whether each material, system or method listed is relevant to your study. If you are not sure if a list item applies to your research, read the appropriate section before selecting a response.

Materials & experimental systems

n/a	Involved in the study	
☐	☒	Antibodies
☐	☒	Eukaryotic cell lines
☒	☐	Palaeontology and archaeology
☐	☒	Animals and other organisms
☒	☐	Clinical data
☒	☐	Dual use research of concern

Methods

n/a	Involved in the study	
☒	☐	ChIP-seq
☒	☐	Flow cytometry
☒	☐	MRI-based neuroimaging

Antibodies

Antibodies used	Primary antibody:<br>1. goat anti-GFAP antibody (Abcam, Cambridge, MA, USA, ab53554)<br>2. rabbit anti-CaMKIIα (Abcam, Cambridge, MA, USA, ab5683)<br>3. mouse anti-GABA (Sigma-Aldrich, St. Louis, MO, USA, A0310)

	Secondary antibody:<br>4. rabbit anti-goat IgG conjugated with Cy3 (The Jackson Laboratory, Bar Harbor, ME, USA, 305-165-003)<br>5. goat anti-rabbit IgG conjugated with Alexa Fluor® 647 (H+L) (The Jackson Laboratory, Bar Harbor, ME, USA, 111-605-003)<br>6. Donkey anti-Mouse Alexa Fluor® 647 IgG (H+L) (The Jackson Laboratory, Bar Harbor, ME, USA, 715-605-151).
Validation	All used antibodies are commercially available. Validation details for primary antibodies can be found at:<br>1. https://www.abcam.com/products/primary-antibodies/gfap-antibody-ab53554.html<br>2. https://www.abcam.com/products/primary-antibodies/camkii-alpha-phospho-t286-antibody-ab5683.html<br>3. https://www.sigmaaldrich.cn/CN/zh/product/sigma/a0310

Eukaryotic cell lines

Policy information about cell lines and Sex and Gender in Research

Cell line source(s)	The HEK293T cell line were purchased from the American Type Culture Collection (Manassas, VA, USA).
Authentication	The HEK293T cell line maintained in the lab and not authenticated for this study.
Mycoplasma contamination	The HEK293T cell line tested negative for mycoplasma contamination.
Commonly misidentified lines<br>(See ICLAC register)	No commonly misidentified lines was used.

Animals and other research organisms

Policy information about studies involving animals; ARRIVE guidelines recommended for reporting animal research, and Sex and Gender in Research

Laboratory animals	8–10 week-old adult male C57BL/6 mice (Hunan SJA Laboratory Animal Company, Changsha, Hunan, China), Ai14 transgenic mice (The Jackson Laboratory, Bar Harbor, ME, USA) and Vgat-ires-Cre transgenic mice (The Jackson Laboratory, Bar Harbor, ME, USA) were used for experiments. Additionally, ten-month-old APP/PS1 (AD) transgenic mice and wild-type (WT) C57BL/6 mice (provided by the Xuzhou Medical University, Xuzhou, Jiangsu, China) were used for detecting circuit changes in Alzheimer's disease model mice. The mice were housed under a 12/12-h light/dark cycle in specific pathogen-free facilities with controlled temperature (22–24 °C) and humidity (40–60%), water and food were supplied ad libitum. All the surgical and experimental procedures were performed following the guidelines formulated by the Animal Care and Use Committee of Innovation Academy for Precision Measurement Science and Technology, Chinese Academy of Sciences.
Wild animals	Not used.
Reporting on sex	Male mice were used in the experiments to avoid the interference of the female estrous cycle. This experiment involves the identification of vector-targeted transduction of neurons or glial cells, which is independent of gender.
Field-collected samples	Not collected.
Ethics oversight	All the surgical and experimental procedures were performed following the guidelines formulated by the Animal Care and Use Committee of Innovation Academy for Precision Measurement Science and Technology, Chinese Academy of Sciences.

Note that full information on the approval of the study protocol must also be provided in the manuscript.
